# Enamel Surface Roughness after Lingual Bracket Debonding: An In Vitro Study

**DOI:** 10.3390/ma12244196

**Published:** 2019-12-13

**Authors:** Martina Eichenberger, Anna Iliadi, Despina Koletsi, George Eliades, Carlalberta Verna, Theodore Eliades

**Affiliations:** 1 Clinic of Orthodontics and Pediatric Dentistry, Center of Dental Medicine, University of Zurich, 8032 Zurich, Switzerland; martina.eichenberger@zzm.uzh.ch (M.E.); d.koletsi@gmail.com (D.K.); 2 Department of Orthodontics and Pediatric Dentistry, Centre for Dental Medicine, University of Basel, Hebelstrasse 3, CH-4056 Basel, Switzerland; carlalberta.verna@unibas.ch; 3 Department of Biomaterials, School of Dentistry, National and Kapodistrian University of Athens, 11527 Athens, Greece; annaeliades@gmail.com (A.I.); geliad@dent.uoa.gr (G.E.)

**Keywords:** enamel roughness, 3D profilometry, lingual orthodontics, debonding

## Abstract

The aim of the present study was to quantitatively assess changes in enamel roughness parameters before and after lingual bracket debonding. The lingual surface of 25 sound premolars extracted for orthodontic reasons was studied by 3D optical interferometric profilometry before and after debonding of lingual brackets following enamel finishing (with fine diamond) and polishing (with 12- and 20-fluted carbide burs). The roughness parameters tested were the amplitude parameters Sa and Sz, the hybrid parameter Sdr, and the functional parameters Sc and Sv. The parameter differences (after debonding-reference) were calculated, and statistical analysis was performed via a Wilcoxon signed-rank test. Statistically significantly higher values were observed in all the surface roughness parameters of enamel surfaces after finishing and polishing, with the mostly affected parameter being the Sdr. Under the conditions of the present study, the finishing and polishing instruments used after debonding of lingual noncustomized brackets created a surface texture rougher than the control in all the tested roughness parameters.

## 1. Introduction

In the last decade, the demand for invisible orthodontics and aesthetic considerations, primarily across adult patients, has been increasing. Various different aesthetic approaches such as ceramic or polycarbonate brackets, thermoplastic aligners, and fixed lingual orthodontic appliances have been developed, the latter being the only literally invisible appliance system available.

Since the introduction of fixed lingual appliances in 1979 by Fujita [1], progress has been made in terms of design, manufacturing, and mechanotherapy. At present, clinicians are able to treat even complex cases with the use of fixed lingual orthodontic appliances. Although evidence regarding lingual fixed orthodontic appliances’ adverse effects is not robust [2], patient discomfort inherent with speech or eating difficulties, tongue irritation, and difficulty in maintaining oral hygiene are usually reported and associated with the insertion of the appliances [2,3,4].

Bonding techniques in lingual brackets may differ slightly from conventional buccal brackets. As access and visibility are compromised, indirect bonding is popular especially among complex procedures, mainly due to the demand for precision in bracket positioning due to the irregular shape of the lingual surface, which strongly influences the spatial orientation of the crown. Direct bonding might also be an option mainly for two-dimensional corrections and sectional appliances [5]. Regardless of the bonding protocol, the procedure is based on enamel acid etching and resin infiltration into the microporosity created, similar to buccal bracket bonding. According to Brosh et al., the etching procedure leads to an extended dissolution of enamel prisms resulting in bigger pores on the buccal side of the teeth when compared to the lingual side. Moreover, higher debonding forces were measured on the buccal side, whereas the adhesive remnant index was higher on the lingual side [6]. After debonding, the mechanical removal of the residual adhesive from the enamel surface may lead to enamel damage, resulting in the increase of surface roughness. A number of studies have evaluated the results of debonding and polishing of buccal enamel surfaces [7,8,9]. In a systematic review evaluating enamel surface roughness after debonding buccal brackets, it was reported that enamel loss and surface roughness depend primarily on the adhesive removal protocol, while secondarily on the pressure exerted against the enamel surface during the procedure [8]. Adequate adhesive removal and tooth polishing is essential, since scratches and cracks remaining on the enamel surface facilitate plaque accumulation and possibly caries development [10].

Tungsten carbide burs in conjunction with a multistep Sof-Lex disc system for polishing are recommended, as they can achieve a smooth surface [8]. Furthermore, for gross adhesive removal, finishing diamond burs may be suitable due to their high cutting efficiency [11]. Tooth morphology on the lingual side differs from the tooth morphology of the buccal side. This is the case not only for the front teeth, but also for premolars, which have different curvatures and a different enamel structure on the lingual side. The untreated lingual surface is less rough, and the perikymata seem to appear to a lesser degree than on the buccal side [6]. These differences have an impact on the dissolution of enamel prisms during etching, which effects bracket bonding. They might also have an impact on the debonding procedure.

To the authors’ knowledge, there is no study evaluating the lingual enamel surface roughness after debonding lingual brackets. The aim of the present study was to quantitatively assess the changes in enamel roughness after the debonding of lingual brackets, finishing, and polishing. The null hypothesis was that the finishing and polishing procedures after debonding do not increase the enamel surface roughness in comparison with the native control.

## 2. Materials and Methods 

### 2.1. Sample Preparation

Twenty-five sound premolars extracted for orthodontic reasons and stored in 0.5% chloramine-T at 8 °C were used in this study. The teeth were free of caries or periodontal problems, with intact enamel and no history of exposure to bleaching agents. The roots were embedded in silicon holders to facilitate handling during bonding and polishing. Each tooth was labelled for identification purposes. The teeth were cleaned with a nonfluoride paste (Clean Polish; Hawe-Neos Dental, Bioggio, Switzerland), rinsed with tap water and air-dried. The central region of each lingual surface was marked with a permanent ink marker (Ø: 4 mm), and the surface morphology was examined under a stereomicroscope (M80, Leica, Wetzlar, Germany) operated under reflected light at 20× magnification.

Thereafter, roughness measurements were obtained from each surface. These regions were acid-etched for 30 s with a 35% phosphoric acid gel (Transbond XT etching gel, 3M Unitek, Monrovia, CA, USA), rinsed with water for 10 s and dried with oil-free and moisture-free compressed air for 5 s. Metal lingual brackets (2D Medium Twin Standard, Forestadent, Pforzheim, Germany) were then bonded with Transbond XT (3M Unitek) on these surfaces by firmly pressing the brackets, removing excess resin with a dental explorer, and light-curing from incisal and cervical directions (45° angle, 10 s each) with an LED unit (Bluephase G2, Ivoclar-Vivadent, Schaan, Liechtenstein) emitting 1200 mW/cm^2^ light intensity at standard mode. After storage in distilled water at 37 °C for a week, all brackets were debonded by a single trained operator using a lingual debonding plier (Hu-Friedy Co, Chicago, Ill, USA). The gross amount of resin remnants was removed using an extra fine finishing diamond (Staddard, Hertfordshire, UK), and the surface located residual resin was finally removed with 12-fluted (Dedeco International Inc, Long Eddy, NY, USA) and 20-fluted (Komet Dental, Lemgo, Germany) tungsten carbide burs attached to an air-rotor handpiece with water spray coolant, until no macroscopically (5×) visible adhesive remnants could be found. The finished and polished surfaces were examined again under a stereomicroscope, and the roughness parameters were determined at the same regions under the same conditions.

Roughness analysis was performed by optical interferometric profilometry. A 3D optical profiler (Wyko NT 1100, Veeco, Santa Barbara, CA, USA) was used under the following conditions: vertical scanning mode, Mirau lens, 20× magnification (231.1 × 303.8 μm^2^ analysis area), tilt correction, 5 µm Gaussian high-pass filter to remove surface waviness, and 0.1 nm (z-axis) and 0.2 µm (x-, y-axis) resolution. The following roughness parameters were tested:

(a) The amplitude parameters Sa (the absolute profile deviation versus the average over a 3D surface) and Sz (the 10-point height over the surface, representing the average difference between the five highest peaks and the five lowest valleys).

(b) The hybrid parameter Sdr (the developed interfacial area ratio, expressed as the percentage of developed area due to surface texture compared to an ideal plane of the same size). 

(c) The functional parameters Sc (the core fluid retention index, describing the volume that a surface would support from 5% to 80% of the bearing ratio) and Sv (the valley retention index, describing the volume the surface would support at the valley zone, 80% to 100% of the bearing ratio).

### 2.2. Statistical Analysis

Normality assumptions were checked through Shapiro–Wilk tests and q–q plots. Due to the non-normal distribution of the residuals, nonparametric statistics were used. Descriptive statistics were used to present actual reference and debonded values as well as differences (Δ = debonded-reference) for roughness parameters. The following parameters were explored: Sa, Sz, Sc, Sv, and Sdr. A Wilcoxon signed-rank test for paired data was used to check similarity between debonded and initial roughness values for each parameter. The level of statistical significance was pre-specified at *p* < 0.05. Statistical analyses were performed with STATA version 15.1 software (Stata Corporation, College Station, Tex, USA).

All analyses were undertaken in Stata version 15.1 software (StataCorp, College Station, Texas, USA).

## 3. Results

**Figure 1:**
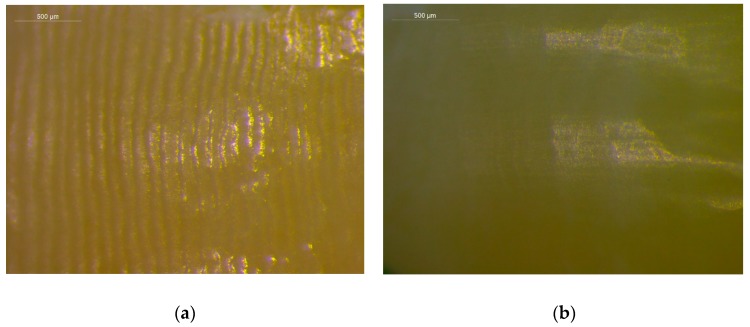
Stereomicroscopic images of intact (**a**) and of the corresponding debonded and polished surfaces (**b**). The intact surfaces demonstrate higher gloss but extensive waviness due to the presence of enamel perikymata. The debonded and polished surfaces are duller, with reduced waviness (reflection mode, bar = 500 μm).

Representative reflected light stereomicroscopic images of intact and polished surfaces after bracket debonding finishing and polishing are illustrated in Figure 1. The intact reference surfaces demonstrated higher specular light reflectivity (gloss). However, the morphology demonstrated excessive waviness attributed to enamel perikymata. The debonded finished and polished surfaces exhibited reduced gloss with less waviness.

**Figure 2:**
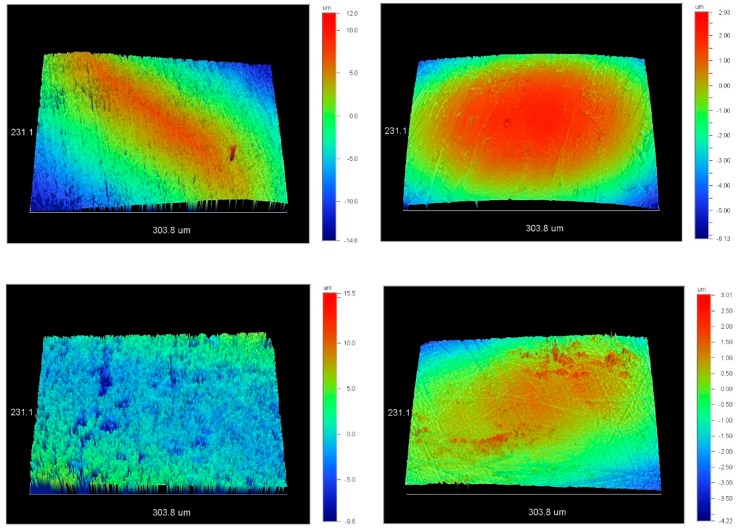
3D profilometric images of intact (left column) and of the corresponding debonded and polished surfaces (right column). Each scale bar represents the highest (red) to lowest (dark blue) probed region (20× magnification, 231.1 × 303.8 μm^2^ area analyzed, bar scale in μm).

Representative 3D profilometric images of the reference and the debonded finished and polished surface are illustrated in Figure 2.

The reference surfaces were smooth with an amplitude deviation ranging from 7 to 9 μm, whereas the corresponding numbers after debonding finishing and polishing ranged between 25 and 27 μm, indicating increased amplitude deviation. The descriptive statistics for surface roughness parameters Sa, Sz, Sc, Sv, and Sdr for the reference and the debonded surfaces separately are presented in Table 1, while the descriptive statistics for the differences (Δ = debonded-reference) are shown in Table 2. The Wilcoxon signed-rank test revealed very strong evidence for a statistically significant difference between intact enamel and debonded surfaces for all assessed roughness parameters (Sa, Sc, Sv, Sdr: *p*-value < 0.001; Sz: *p*-value = 0.004). The most thoroughly affected parameter was Sdr with a 1.7× increase, whereas all the other parameters ranged from a 1.2× to 1.4× increase after debonding, finishing, and polishing.

## 4. Discussion

Enamel reduction that might occur during debonding may compromise tooth resistance, creating enamel cracks and fractures in combination with increasing surface roughness [12,13]. This may lead to plaque accumulation resulting in caries development. Adequate finishing and polishing of the debonded surfaces is substantial in order to preserve the enamel surface [14].

The measured roughness parameters of natural surfaces may be influenced by the measurement device and the magnification. Only a quantitative assessment of roughness parameters allows for direct comparisons between treated and nontreated enamel surfaces. Scanning electron microscopy (SEM) is unreliable and subjective, and the surfaces cannot be quantitatively evaluated. 3D noncontact optical profilometry is superior to the contact surface roughness measuring devices, as 3D noncontact optical devices are not dependent on the stylus tip diameter [8]. In addition, 3D noncontact optical profilometry is nondestructive. Therefore, measurements can be taken on the same specimens and at the same region before bonding (reference) and after debonding and polishing. In the present study, the lingual enamel surface roughness after debonding lingual brackets was investigated using 3D noncontact optical profilometry.

The null hypothesis was rejected as all enamel surface roughness parameters (Sa, Sz, Sv, Sc, and Sdr) were significantly higher after debonding and polishing. The statistically significant difference found in the amplitude parameters Sa and Sz implies that the enamel surface after debonding and polishing has higher peaks and/or deeper valleys compared to the reference enamel surface.

A number of studies have examined the buccal enamel surface roughness after debonding buccal brackets analyzing different debonding and polishing protocols [7,9,15,16,17,18,19,20,21]. 3D noncontact optical profilometry was also used in the study of Ferreira et al. [22]. They analyzed the buccal enamel surfaces after debonding buccal brackets. As they did not use etching and based their bonding procedures on inherently different bonding materials, such as resin-modified glass ionomer cements, their enamel loss and roughness parameters may be substantially different from the values identified in the present study. Several other studies using similar bonding agents have followed different methods analyzing the surface roughness, such as atomic force microscopy [17] or contact optical profilometry [7,16,20]. Due to dissimilarities in the methodologies described, it is difficult to directly compare the results of the present study with those of other studies.

None of the existing studies evaluated the lingual enamel surface. There might be some differences between the debonding procedure on lingual and buccal enamel surface. Wang et al. showed similar bond strength on lingual and buccal surfaces of premolars [23], but Brosh et al. found a significantly higher bonding strength on the buccal side [6]. The bonding strength of lingual brackets depends on the design of the bracket base. Due to their larger bases, customized metal bracket have relatively high bond strength values [24]. In the present study, noncustomized 2D brackets were used. Sfondrini et al. showed that noncustomized brackets have similar bonding values as buccal brackets [25]. However, the adhesive remnant index (ARI) after debonding noncustomized lingual brackets seems to be higher than the ARI after debonding buccal brackets [25]. Therefore, increased amounts of residual adhesive have to be removed on the lingual side. Several protocols for resin removal exist. The methods of the present study included the use of a fine finishing diamond and 12- and 20-bladed tungsten carbide burs. Eliades et al. compared the buccal enamel surface roughness after two different resin removal methods [7]. The 8-bladed tungsten carbide bur was superior to the ultrafine diamond bur regarding the enamel roughness. However, the ultrafine diamond bur was more time-saving. In the present study, a combination of the two methods was applied. With the finishing diamond, the gross amount of adhesive remnants was efficiently removed without touching the enamel. The surface-located adhesive was removed with tungsten carbide burs, which is the most common method for adhesive removal [8]. The preliminary use of ultrafine diamond burs before using carbide burs for grinding adhesives was also recommended by Jung [11]. Debonding of brackets bonded to enamel involves a wide range of often empirically derived measures and steps, and there is no universally-suggested or implemented standard protocol. Sof lex discs, for example, may be suggested for the finishing stages of the process, although these can only result in the polishing of the adhesive remnants on the enamel through mechanical and thermal treatment of the resin, which results in lower variation of the surface profile.

In the present study, the analyzed area was standardized to a preliminary limited size of a 4 mm diameter. When debonding lingual brackets, the extension of the grinded and damaged area depends essentially on the bracket design. As such, brackets with customized metal bracket bases may lead to a more extensive ground enamel areas due to their increased basal profile. On top of the usual careful consideration on patient selection in conjunction with individual oral hygiene characteristics, clinical procedures that allow better the identification of bonding materials at debonding should be considered [10,26,27,28]. The limitations pertain to the fact that there is a lack of an aging process involved in the protocol, which could have some implications in altering the extent of results in either direction (alleviating or enhancing them).

## 5. Conclusions

Debonding lingual brackets from enamel results in an increase of a number of roughness parameters. The most affected parameter was Sdr, associated with the enamel surface area exposed. Depending on the type of lingual bracket type used, this increased roughness may involve a significant area of enamel surface with the potential of enhancing the plaque-retaining capacity of teeth.

## Figures and Tables

**Table 1:** Descriptive statistics of reference and debonded values for roughness parameters (n = 25).

Roughness Parameters					
	Sa (nm)	Sz (μm)*	Sc (nm^3^/nm^2^)	Sv (nm^3^/nm^2^)	Sdr (%)
**Reference**					
Median	41.38	1.09	64.41	8.88	2.49
Interquantile Range (25–75 percentile)	25.99–50.01	0.89–1.31	33.53–78.19	5.82–10.07	0.95–3.40
Minimum	14.34	0.78	19.84	3.00	0.41
Maximum	74.57	2.35	110.01	16.11	6.77
**Debonded**					
Median	59.19	1.35	88.09	10.96	4.18
Interquantile Range (25–75 percentile)	54.71–63.75	1.22–1.72	78.80–94.48	10.44–11.64	3.71–4.64
Minimum	51.76	1.17	73.09	9.82	3.38
Maximum	157.60	5.27	229.09	33.36	35.30

* Two strong outliers were excluded for this parameter (n = 23).

**Table 2:** Descriptive statistics of differences (Δ = debonded-reference) between roughness parameters in terms of debonded and initial reference values (n = 25).

Roughness Parameters			
Median	Interquantile Range	Minimum	Maximum
***ΔSa*** (nm)			
17.16	38.02	−19.40	134.68
***ΔSz*** (μm)*			
0.20	0.56	−1.02	4.38
***ΔSc*** (nm^3^/nm^2^)			
25.03	55.91	−31.67	199.1
***ΔSv*** (nm^3^/nm^2^)			
2.43	5.62	−5.15	28.05
***ΔSdr*** (%)			
1.79	3.27	−3.02	34.56

* Two strong outliers were excluded for this parameter (n = 23).
